# Systematic Scoring of Tubular Injury Patterns Reveals Interplay between Distinct Tubular and Glomerular Lesions in ANCA-Associated Glomerulonephritis

**DOI:** 10.3390/jcm10122682

**Published:** 2021-06-18

**Authors:** Samy Hakroush, Désirée Tampe, Peter Korsten, Philipp Ströbel, Björn Tampe

**Affiliations:** 1 Institute of Pathology, University Medical Center Göttingen, 37075 Göttingen, Germany; samy.hakroush@med.uni-goettingen.de (S.H.); philipp.stroebel@med.uni-goettingen.de (P.S.); 2 Department of Nephrology and Rheumatology, University Medical Center Göttingen, 37075 Göttingen, Germany; desiree.tampe@med.uni-goettingen.de (D.T.); peter.korsten@med.uni-goettingen.de (P.K.)

**Keywords:** systemic vasculitis, autoimmune diseases, anti-neutrophil cytoplasmic antibody, ANCA glomerulonephritis, acute tubular injury

## Abstract

Background: Antineutrophil cytoplasmic antibody (ANCA)-associated vasculitis (AAV) is a small vessel vasculitis, most frequently presenting as microscopic polyangiitis (MPA) or granulomatosis with polyangiitis (GPA). Acute tubular injury with the presence of tubulitis was previously reported to be of prognostic value in ANCA glomerulonephritis (GN). In particular, distinct tubular injury lesions were associated with the deterioration of kidney function at AAV disease onset, as well as renal resistance to treatment, and higher risk of progression to composite outcome in patients with AAV. To expand our knowledge regarding distinct tubular lesions in AAV, we aimed to describe acute tubular injury patterns in association with glomerular lesions in ANCA GN by systematic histological scoring. Methods: A total number of 48 renal biopsies with confirmed renal involvement of AAV admitted to the University Medical Center Göttingen from 2015 to 2020 were retrospectively examined. By systematic scoring of tubular injury lesions, the association between clinical parameters, laboratory markers, and histopathological findings was explored. Results: We have shown that cellular casts in renal biopsies were frequently observed in the majority of cases with ANCA GN. Furthermore, we showed that tubular epithelial simplification with dilatation correlated with MPA and MPO subtypes, C3c hypocomplementemia, severe renal involvement, and uACR. Red blood cell (RBC) casts were associated with increased levels of C-reactive protein (CRP), leukocyturia, and hematuria. Finally, we found that hyaline casts were associated with an increased fraction of glomeruli with global glomerular sclerosis. Conclusions: Acute tubular injury patterns were correlated with active ANCA GN, whereas tubular injury lesions reflecting the later stages of kidney disease correlated with chronic glomerular lesions. These results suggest an interplay between different renal compartments.

## 1. Introduction

Antineutrophil cytoplasmic antibody (ANCA)-associated vasculitis (AAV) is a small vessel vasculitis that, according to the 2012 revised Chapel Hill Consensus Conference Nomenclature of Vasculitides, most frequently presents as microscopic polyangiitis (MPA) or granulomatosis with polyangiitis (GPA) [1,2]. Renal involvement is a common and severe complication of AAV, potentially resulting in a pauci-immune necrotizing and crescentic ANCA glomerulonephritis (GN) with acute kidney injury (AKI), end-stage renal disease (ESRD), or death [2]. Clinicopathologic studies from the European Vasculitis Study Group (EUVAS) have demonstrated that distinct glomerular lesions are related to renal outcome in ANCA GN [3,4,5,6]. Derived from these studies, a histopathological subgrouping into four classes (focal, crescentic, mixed, and sclerotic), as defined by Berden et al., was shown to predict long-term renal survival rates [7]. These results were confirmed in multiple independent studies in recent years [8,9,10,11,12,13,14,15,16,17,18,19,20,21,22,23,24]. However, multivariable analyses could not demonstrate any improvement of outcome prediction in most of these studies, mainly attributed to the lack of outcome difference in the crescentic and mixed classes [16,17,18,19,20,21,22,23,24,25,26,27]. Therefore, Brix et al. suggested the ANCA renal risk score (ARRS) through the incorporation of tubular atrophy/interstitial fibrosis (TA/IF) to the percentage of normal glomeruli and baseline glomerular filtration rate (GFR) to predict ESRD in patients with AAV, underscoring the relevance of tubulointerstitial and glomerular lesions in ANCA GN [28]. Furthermore, acute tubular injury with the presence of tubulitis was previously reported to be of prognostic value in ANCA GN [4,5,29]. In particular, distinct tubular injury lesions were associated with the deterioration of kidney function at AAV disease onset, renal resistance to treatment, and a higher risk of progression to composite outcome in patients with AAV [30]. As a result of persistent tubular injury, TA/IF develops and further impairs kidney function, being characterized by a vicious circle of tubular epithelial damage, TA/IF, and chronic glomerular injury [31,32]. To expand our knowledge of distinct histomorphological tubular changes in AAV, we aimed to describe tubular injury lesions in association with glomerular lesions in ANCA GN by systematic histological scoring [33].

## 2. Materials and Methods

### 2.1. Study Population

A total number of 48 renal biopsies with confirmed renal involvement of AAV admitted to the University Medical Center Göttingen from 2015 to 2020 were retrospectively examined. The patient cohort has, in part, been previously described [34,35,36,37]. Medical records were used to obtain data on the age, sex, diagnosis (MPA or GPA), and laboratory results. The estimated glomerular filtration rate (GFR) was calculated using the Chronic Kidney Disease Epidemiology Collaboration (CKD-EPI) equation [38]. The worst measurement during the initial course of the disease was used to determine the severity of kidney injury.

### 2.2. Definitions

At admission, the Birmingham Vasculitis Activity Score (BVAS) version 3 was calculated as described previously [39]. The BVAS is assessed on a scale from 0 to 63, with a score of 0 indicating the absence of disease activity and higher scores indicating active disease. The simplified acute physiology score (SAPS) II was calculated according to the published guidelines [40]. RRT was performed intermittently in all cases, indications of RRT included serum creatinine ≥5.8 mg/dL (≥500 μmol/L), severe electrolyte or acid-base abnormalities, and volume overload or uremic encephalopathy. RRT was terminated when the GFR surpassed 15 mL/min/1.73 m^2^ in the absence of hyperkalemia, heart failure, edema, or uremic encephalopathy. 

### 2.3. Urinary Analysis

Levels of total proteinuria, urinary albumin, immunoglobulin G (IgG), α_1_-microglobulin, and α_2_-macroglobulin were normalized to urinary creatinine concentration to control for variations in urine flow rate [41]. Leukocyturia and hematuria per high-power field (HPF) were semiquantitatively scored into 0: negative, 1: 2–4/HPF, 2: 5–9/HPF, 3: 10–20/HPF and 4: >20/HPF. Acanthocytes were scored for presence/absence. 

### 2.4. Renal Histopathology

Two renal pathologists (SH and PS) independently evaluated the kidney biopsies and were blinded to data analysis. Within a renal biopsy specimen, each glomerulus was scored separately for the presence of necrosis, crescents and global glomerular sclerosis. Consequently, the percentage of glomeruli with any of these features was calculated as a fraction of the total number of glomeruli in each renal biopsy. Based on these scorings, histopathological subgroupings according to Berden et al. (focal, crescentic, mixed or sclerotic class), and ARRS according to Brix et al. (low, medium or high risk), were performed [7,28]. Systematic histological scoring of tubular injury lesions was evaluated as previously described [33]. In brief, epithelial simplification and tubular dilatation, nonisometric cell vacuolization, cellular, red blood cell (RBC), and hyaline casts were given a score between 0 and 4 as a percentage of the total affected cortical area of the biopsy (score 0: <1%, 1: ≥1–10%, 2: ≥10–25%, 3: ≥25–50%, 4: >50%). Kidney biopsies were also evaluated analogous to the Banff scoring system for allograft pathology [42]. Banff score lesions included interstitial inflammation (*i*), tubulitis (*t*), arteritis (*v*), glomerulitis (*g*), interstitial fibrosis (*ci*), tubular atrophy (*ct*), arteriolar hyalinosis (*ah*), peritubular capillaritis (*ptc*), total inflammation (*ti*), inflammation in areas of IFTA (*i-IFTA*), and tubulitis in areas of IFTA (*t-IFTA*). The Banff scoring system had three grades: none (0), mild (1), moderate (2), and severe (3). The cut-off points for *i* were <10%, 10–25%, 26–50% and >50%, respectively. Cut-off points for *t* were 0, 1–4, 5–10 and >10 mononuclear cells/tubular cross section. Cut-off points for *v* were no arteritis, mild- to-moderate intimal arteritis in at least 1 arterial cross section, severe intimal arteritis with at least 25% luminal area lost in at least 1 arterial cross section, transmural arteritis and/or arterial fibrinoid change, and medial smooth muscle necrosis with lymphocytic infiltrate in vessel, respectively. Cut-off points for *g* were no glomerulitis, segmental or global glomerulitis in less than 25% of glomeruli, segmental or global glomerulitis in 25 to 75% of glomeruli, and segmental or global glomerulitis in more than 75% of glomeruli. Cut-off points for *ci* were interstitial fibrosis in up to 5%, 6–25%, 26–50% and >50% of cortical area. Cut-off points for *ct* were no tubular atrophy, and tubular atrophy involving up to 25%, 26–50% and >50% of the area of cortical tubules. Cut-off points for *ah* were no PAS-positive hyaline arteriolar thickening, mild-to-moderate PAS-positive hyaline thickening in at least 1 arteriole, in more than 1 arteriole, and in many arterioles. Cut-off points for *ptc* were a maximum number of leukocytes <3, at least 1 leukocyte cell in ≥10% of cortical peritubular capillaries (PTCs) with 3–4 leukocytes in most severely involved PTC, at least 1 leukocyte in ≥10% of cortical PTC with 5–10 leukocytes in most severely involved PTC, and at least 1 leukocyte in ≥10% of cortical PTC with >10 leukocytes in most severely involved PTC. Cut-off points for *ti* were <10%, 10–25%, 26–50% and >50% of total cortical parenchyma inflamed. Cut-off points for *i-IFTA* and *t-IFTA* were no inflammation or less than 10%, 10–25%, 26–50% and >50% of scarred cortical parenchyma.

### 2.5. Statistical Methods

Variables were tested for normal distribution using the Shapiro–Wilk test. Non-normally distributed continuous variables are expressed as median and interquartile range (IQR), and categorical variables are presented as frequency and percentage. Statistical comparisons were not formally powered or prespecified. For group comparisons, the Mann–Whitney U-test was used to determine differences in medians. Nonparametric between-group comparisons were performed with Pearson’s Chi-square test. Spearman’s correlation was performed to assess the correlation between clinical, laboratory, and histopathological parameters, and heatmaps reflecting the mean values of Spearman’s ρ are shown, the asterisks indicating significant correlations. Data analyses were performed with GraphPad Prism (version 9.1.1 for MacOS, GraphPad Software, San Diego, CA, USA).

## 3. Results

### 3.1. Description of Study Population

**Figure 1:**
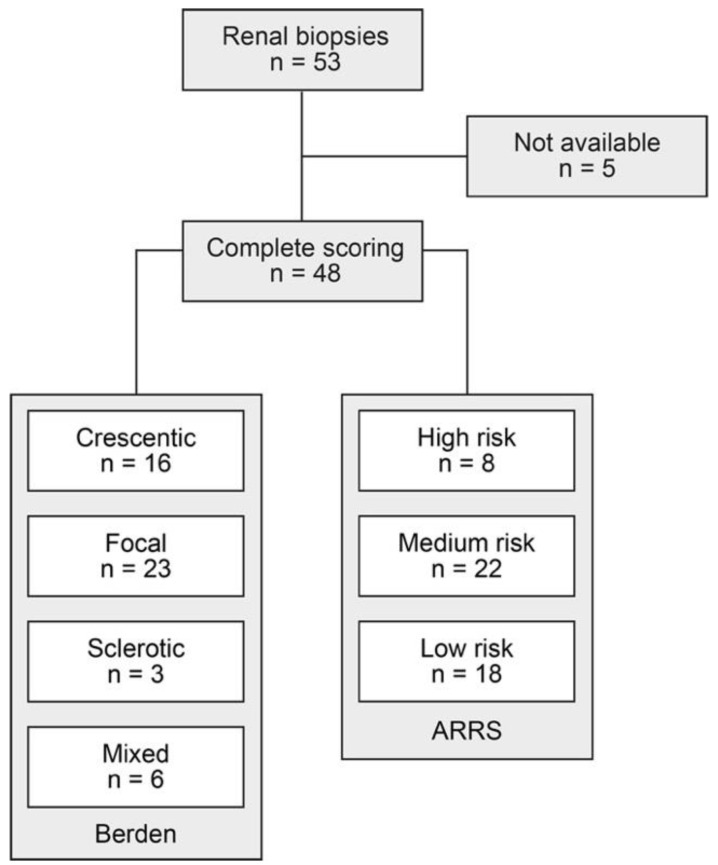
Total patient cohort of ANCA GN: STROBE flow chart of patient disposition with systematic histological scoring of tubular injury lesions. Abbreviations: ANCA, anti-neutrophil cytoplasmic antibodies; ARRS, ANCA renal risk score; GN, glomerulonephritis; STROBE, Strengthening the Reporting of Observational Studies in Epidemiology.

A total number of 53 renal biopsies with confirmed renal involvement of AAV were identified. Among them, complete systematic histological scoring of tubular injury lesions was available for 48/53 (90.6%) renal biopsies (Figure 1). Histopathological subgroupings revealed 16/48 (33.3%) crescentic, 23/48 (47.9) focal, 3/48 (6.3%) sclerotic, and 6/48 (12.5%) mixed class ANCA GN [7]. ARRS was high in 8/48 (16.7%), medium in 22/48 (45.8%), and low risk class ANCA GN in 18/48 (37.5%) cases (Figure 1) [28].

The baseline characteristics of the entire cohort are shown in Table 1. In this cohort, the median (IQR) age at diagnosis was 63 (54.25–74) years, 19/48 (39.6) were female, and all were Caucasian. Disease onset was 18 (7–46) days before admission, and kidney biopsy was performed 6 (3–9.5) days after admission to confirm the renal involvement of AAV. There were 26/48 (54.2%) positive for myeloperoxidase (MPO) and 22/48 (45.8%) positive for proteinase 3 (PR3) ANCA. There were 26/48 (54.2%) patients categorized as MPA and the remainder as GPA. The majority of patients (85.4%) had a new diagnosis of AAV. There were 39/48 (81.3) patients with extrarenal manifestation of AAV (28 with lung, 8 with sinus, 10 with joint, 4 with ear, 3 with eye, 5 with peripheral nerve, and 8 with skin involvement), and 5/48 (10.4%) had alveolar hemorrhage. The worst median (IQR) eGFR at disease onset was 17.25 (9.525–48.15) mL/min/1.73 m^2^, and 16/48 (33.3) required dialysis within 30 days after admission. Tubular injury lesions were detectable in a total number of 46/48 (95.8%) ANCA GN cases (Table 1).

**Figure 2:**
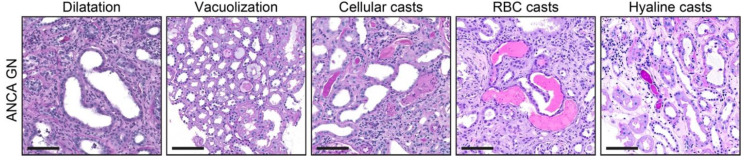
Tubular injury lesions in ANCA GN: Representative photomicrographs with tubular injury lesions are shown (scale bars: 100 μm). Abbreviations: ANCA, anti-neutrophil cytoplasmic antibodies; GN, glomerulonephritis; RBC, red blood cell.

Systematic histological scoring of tubular changes revealed that tubular epithelial simplification and dilatation was present in 44/48 (91.7%) of ANCA GN, epithelial vacuolization in 9/48 (18.8%), cellular casts in 19/48 (60.4%), red blood cell (RBC) casts in 21/48 (43.8%), and hyaline casts in 28/48 (58.3%) of cases (Figure 2 and Table 1), with variable extent of affected cortical area of the biopsy (score 0: <1%, 1: ≥1–10%, 2: ≥10–25%, 3: ≥25–50%, 4: >50%, Table 2). In summary, tubular injury lesions in renal biopsies were frequently observed in the majority of cases, confirming that the systematic histological scoring of tubular injury lesions, as described previously, is also applicable in ANCA GN [33].

**Figure 3:**
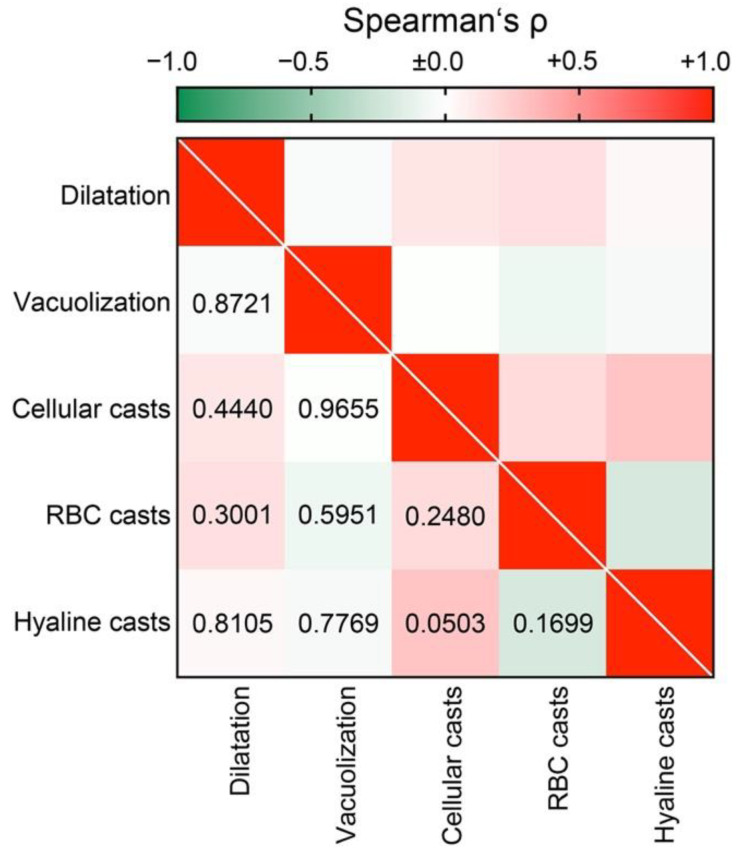
Direct association between tubular injury lesions in AAV: Association between distinct tubular injury lesions are shown by heatmap reflecting mean values of Spearman’s ρ, values of *p* are shown for each association. Abbreviations: AAV, ANCA-associated vasculitis; RBC, red blood cell.

To elucidate the association between tubular injury lesions in ANCA GN, we next analyzed the association of distinct lesions among each other. Interestingly, we did not find any association between different tubular injury lesions (Figure 3), implicating that presence of each lesion reflected a distinct tubular injury. 

### 3.2. Distinct Clinical Parameters and Laboratory Markers Associate with Tubular Injury Lesions in AAV

**Figure 4:**
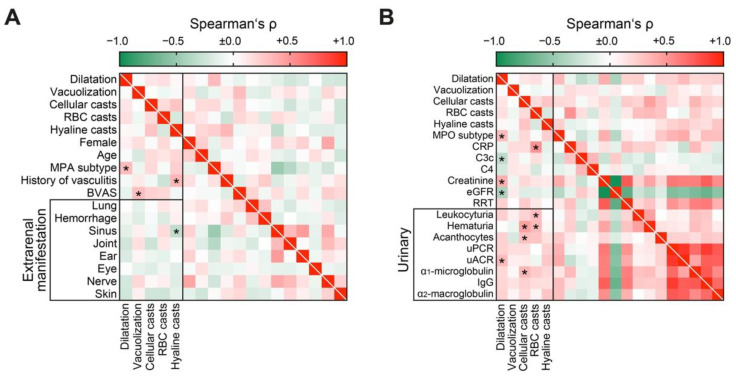
Distinct clinical parameters and laboratory markers associate with tubular injury lesions in AAV: (**A**) Association between tubular injury lesions and clinical parameters are shown by heatmap reflecting mean values of Spearman’s ρ, asterisks indicate *p* < 0.05. (**B**) Association between tubular injury lesions and laboratory markers are shown by heatmap reflecting mean values of Spearman’s ρ, asterisks indicate *p* < 0.05. Abbreviations: AAV, ANCA-associated vasculitis; BVAS, Birmingham Vasculitis Activity Score; C3c, complement factor 3 conversion product; C4, complement factor 4; CRP, C-reactive protein; eGFR, glomerular filtration rate (CKD-EPI); GN, glomerulonephritis; IgG, immunoglobulin G; MPA, microscopic polyangiitis; MPO, myeloperoxidase; RBC, red blood cell; RRT, renal replacement therapy; uACR, urinary albumin-to-creatinine ratio, uPCR, urinary protein-to-creatinine ratio.

We first analyzed tubular injury lesions in association with clinical parameters, laboratory systemic, and urinary markers in AAV. Tubular epithelial simplification and dilatation correlated with the MPA subtype, epithelial vacuolization with systemic disease activity reflected by BVAS not attributed to a specific extrarenal manifestation, and hyaline casts with a history of AAV and less sinus involvement (Figure 4A). Among systematic laboratory parameters, tubular dilatation associated with MPO subtype, C3c hypocomplementemia, severe renal involvement, and urinary albumin-to-creatinine ratio (uACR, Figure 4B). Cellular casts correlated with hematuria, detection of urinary acanthocytes, and tubular proteinuria reflected by urinary α_1_-microglobulin (Figure 4B). Finally, RBC casts were associated with increased levels of C-reactive protein (CRP), leukocyturia, and hematuria (Figure 4B), implicating that RBC casts represent active glomerular disease and subsequent glomerular hemorrhage. In summary, systematic scoring of tubular injury patterns revealed distinct clinical parameters and laboratory markers in association with tubular lesions in AAV.

### 3.3. Acute Tubular Injury Patterns Correlate with Active ANCA GN and Tubular Lesions Reflecting Later Stages of Kidney Disease Correlated with Chronic Glomerular Lesions

**Figure 5:**
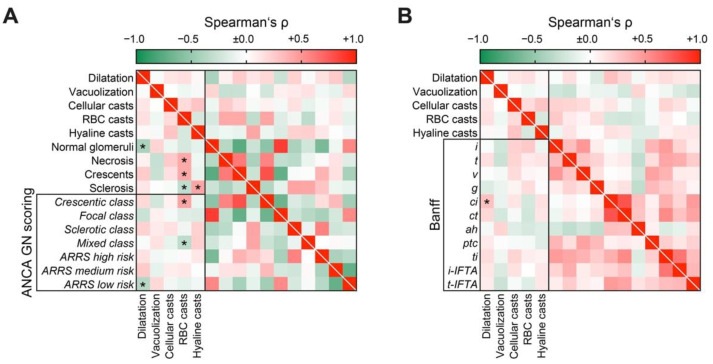
Acute tubular injury patterns correlate with active ANCA GN, whereas tubular lesions reflecting later stages of kidney disease correlated with chronic glomerular lesions: (**A**) Association between tubular injury lesions and histopathological, findings are shown by heatmap reflecting mean values of Spearman’s ρ, asterisks indicate *p* < 0.05. (**B**) Association between tubular injury and interstitial lesions analogous to the Banff scoring system are shown by heatmap reflecting mean values of Spearman’s ρ, asterisks indicate *p* < 0.05. Abbreviations: *ah*, arteriolar hyalinosis; ANCA, anti-neutrophil cytoplasmic antibodies; *ci*, interstitial fibrosis; *ct*, tubular atrophy; *g*, glomerulitis; GN, glomerulonephritis; *i*, interstitial inflammation; *i-IFTA*, inflammation in IFTA; *t*, tubulitis; *ptc*, peritubular capillaritis; RBC, red blood cell; *ti*, total inflammation; *t-IFTA*, tubulitis in IFTA; *v*, intimal arteritis.

Next, we directly correlated the tubular injury lesions with the histopathological findings in ANCA GN, including glomerular and tubulointerstitial injury. Tubular dilatation was associated with a reduced number of normal glomeruli and ARRS low risk categorization (Figure 5A). RBC casts correlated with active glomerular disease, reflected by necrosis, crescents, and a reduced fraction of glomeruli affected by global glomerular sclerosis (Figure 5A), further corroborating that RBC casts represent active glomerular disease. This was also reflected by categorization into the less mixed class, and was predominant in the crescentic class ANCA GN (Figure 5A). In contrast, hyaline casts were associated with an increased fraction of glomeruli with global glomerular sclerosis (Figure 5A). Interestingly, no interstitial lesion, despite renal fibrosis, correlated with any pattern of tubular injury, implicating that distinct tubular lesions reflected nephron damage, rather than interstitial injury (Figure 5B). In summary, acute tubular injury patterns correlated with active ANCA GN, whereas tubular lesions reflecting later stages of kidney disease correlated with chronic glomerular lesions.

## 4. Discussion

Acute tubular injury was characterized by distinct histological changes, with the loss of brush borders, cytoplasmic vacuolization, and fragmentation with detachment of tubular cells into the lumen producing obstructive casts [43,44]. Finally, severe tubular injury also contributed to epithelial cell apoptosis and necrosis [45]. As a result of persistent tubular injury, TA/IF develops and further impairs kidney function, characterized by a vicious circle of tubular epithelial damage, TA/IF, and chronic glomerular injury [31,32]. Elucidating the molecular alterations that occur in association with distinct tubular injury lesions are of great importance to understand the mechanisms that contribute to acute and chronic kidney damage. Therefore, the determination of the severity, type, and temporal context of injury can also determine molecular responses and kidney damage. Types of injury determines damage to distinct compartments in the kidney, including different nephrons or even segments [46]. In addition, a type of injury can mediate the same or different transcriptional alterations depending on the injured compartment or cellular entity [47]. Therefore, a systematic analysis of injuries to different renal compartments, and their association among each other, is crucial to identify the interplay in response to kidney injury. 

We have expanded our current knowledge of distinct tubular injury lesions by systematic scoring of tubular, glomerular, and interstitial lesions in ANCA GN. We have confirmed that tubular injury lesions in renal biopsies are frequently observed in the majority of cases with ANCA GN. Furthermore, a systematic histological scoring of tubular injury patterns revealed distinct clinical parameters and laboratory markers in association with tubular lesions in AAV. Finally, acute tubular injury patterns correlated with active ANCA GN, reflected by glomerular necrosis and crescents, whereas tubular lesions reflecting later stages of kidney disease correlated with chronic glomerular lesions, reflected by global glomerular sclerosis. We have shown that tubular epithelial simplification with dilatation correlated with MPA and MPO subtypes, C3c hypocomplementemia, severe renal involvement, and uACR. This observation is of interest since MPA and MPO subtypes have previously been associated with more severe acute and chronic renal damage [35,48]. Tubular dilatation is observed after severe kidney injury and has initially been attributed to tubular obstruction [49,50,51]. However, more recent studies revealed that tubular dilatation occurs in the recovery phase of kidney injury, independent of tubular obstruction [52]. Hyperplasia of regenerating, dividing tubular epithelial cells and abnormalities of the tubular basement membrane, contributes to tubular dilatation during kidney repair, finally resolving after kidney repair [52]. Our observations that tubular dilatation correlated with severe kidney injury was in line with this concept. However, we also observed that tubular dilatation was associated with a reduced fraction of normal glomeruli and interstitial fibrosis. In this context, dilated tubules, along with interstitial fibrosis, have previously been considered as evidence of advanced stages of kidney disease [53,54]. Furthermore, we have shown that RBC casts were associated with increased levels of C-reactive protein (CRP), leukocyturia, and hematuria, confirming the concept that RBC casts represent active glomerular disease and subsequent glomerular hemorrhage [55]. This is further supported by the correlation between RBC casts and active glomerular disease reflected by necrosis, crescents, and a reduced fraction of glomeruli affected by global glomerular sclerosis. RBC casts are long known, and have been described in various kidney diseases [56]. In patients with systemic vasculitis, RBC casts due to glomerular hemorrhage are considered a marker of active renal disease [55]. Erythrocytes that pass through the glomerular basement membrane gaps are merged with uromodulin produced in the Henle’s loop and from RBC casts that are excreted into urine [57]. Finally, we found that hyaline casts were associated with an increased fraction of glomeruli with global glomerular sclerosis. Hyaline casts reflect nephron obstruction in the final stage of tubular degeneration, due to renal stasis or nephron obstruction [57]. This concept was further supported by our observation that no interstitial lesion, despite renal fibrosis, correlated with any acute tubular injury pattern, implicating that distinct tubular lesions reflect nephron damage, rather than interstitial injury. 

The main limitations of our study were its retrospective design, the small patient number, and lack of data on long-term renal survival. Nevertheless, we have provided a systematic, histological analysis of tubular injury lesions in ANCA GN frequently observed in the majority of cases with AAV, and have provided evidence for an interplay between different renal compartments.

## 5. Conclusions

To our knowledge, this is the first report of systematic scoring of acute tubular injury patterns in ANCA GN. Acute tubular injury patterns correlated with active ANCA GN, whereas tubular lesions reflecting later stages of kidney disease correlated with chronic glomerular lesions.

## Figures and Tables

**Table 1:** Clinical and laboratory parameters of the total ANCA GN cohort.

Parameter	Value
Median age (IQR)—years	63 (54.25–74)
Female sex—no. (%)	19 (39.58)
Disease onset—days before admission (IQR)	18 (7–46)
Kidney biopsy—days after admission (IQR)	6 (3–9.5)
ANCA subtype MPA/GPA—no. (%)	26 (54.2)/22 (45.8)
History of vasculitis—no. (%)	7 (14.6)
Dialysis within 30 days after admission—no. (%)	16 (33.3)
Median BVAS (IQR)—points	18 (15–20.75)
Extrarenal manifestation—no. (%)	39 (81.3)
Lung involvement—no. (%)	28 (58.3)
Pulmonary hemorrhage—no. (%)	5 (10.4)
Sinus involvement—no. (%)	8 (16.7)
Joint involvement—no. (%)	10 (20.8)
Ear involvement—no. (%)	4 (8.3)
Eye involvement—no. (%)	3 (6.3)
Nerve involvement—no. (%)	5 (10.4)
Skin involvement—no. (%)	8 (16.7)
ANCA subtype MPO/PR3—no. (%)	26 (54.2)
Serum creatinine (IQR)—mg/dL	3.155 (1.355–5.138)
eGFR (IQR)—mL/min/1.73 m^2^	17.25 (9.525–48.15)
CRP (IQR)—mg/L	63.7 (22.38–108)
C3c (IQR)—g/L	1.295 (1.035–1.408)
C4 (IQR)—g/L	0.26 (0.2–0.3075)
uPCR (IQR)—mg/g	1067 (523.6–2038)
uACR (IQR)—mg/g	451.4 (192.4–979.1)
α_1_-microglobulin (IQR)—mg/g	69.67 (34.85–186)
α_2_-macroglobulin (IQR)—mg/g	5.055 (2.903–12.42)
IgG (IQR)—mg/g	56.52 (21.63–209.5)
Leukocyturia (IQR)—per HPF	3 (2–4)
Hematuria (IQR)—per HPF	4 (3.25–4)
Acanthocytes—no. (%)	7 (14.6)
Crescentic class—no. (%)	16 (33.3)
Focal class—no. (%)	23 (47.9)
Sclerotic class—no. (%)	3 (6.3)
Mixed class—no. (%)	6 (12.5)
ARRS high risk—no. (%)	8 (16.7)
ARRS medium risk—no. (%)	22 (45.8)
ARRS low risk—no. (%)	18 (16.7)
Any lesion—no. (%)	46/48 (95.8)
Tubular dilatation—no. (%)	44/48 (91.7)
Vacuolization—no. (%)	9/48 (18.8)
Cellular casts—no. (%)	19/48 (39.6)
RBC casts—no. (%)	21/48 (43.8)
Hyaline casts—no. (%)	28/48 (58.3)

Continuous variables are expressed as median and interquartile range (IQR), categorical variables are presented as frequency and percentage. Abbreviations: ANCA, anti-neutrophil cytoplasmic antibodies; BVAS, Birmingham Vasculitis Activity Score; C3c, complement factor 3 conversion product; C4, complement factor 4; CRP, C-reactive protein; eGFR, estimated glomerular filtration rate (CKD-EPI); GN, glomerulonephritis; GPA, granulomatosis with polyangiitis; IQR, interquartile range; MPA, microscopic polyangiitis; MPO, myeloperoxidase; no., number; PR3, proteinase 3; RBC, red blood cell; uPCR, urinary protein-to-creatinine ratio; uACR, urinary albumin-to-creatinine ratio.

**Table 2:** Systematic scoring of tubular injury lesions in the total ANCA GN cohort.

Tubular Injury Lesion	0	1	2	3	4
Tubular dilatation—no. (%)	4 (8.3)	7 (14.6)	11 (22.9)	10 (20.8)	16 (33.3)
Vacuolization—no. (%)	39 (81.3)	9 (18.8)	0 (0)	0 (0)	0 (0)
Cellular casts—no. (%)	19 (39.6)	24 (50)	4 (8.3)	0 (0)	1 (2.1)
RBC casts—no. (%)	27 (56.3)	17 (35.4)	4 (8.3)	0 (0)	0 (0)
Hyaline casts—no. (%)	20 (41.7)	21 (43.8)	6 (12.5)	1 (2.1)	0 (0)

Abbreviations: ANCA, anti-neutrophil cytoplasmic antibodies; GN, glomerulonephritis; no., number; RBC, red blood cell.

## Data Availability

Deidentified data are available on reasonable request from the corresponding author.
